# Middle ear squamous papilloma: report of a case and literature review

**DOI:** 10.1016/S1808-8694(15)31344-6

**Published:** 2015-10-20

**Authors:** Samir Cahali, Flávia B. da Silva, Márcia C. Machado, Danielle A. da Silva, Olga M.R. Reforeme, Michel B. Cahali

**Affiliations:** ^1^ Ph.D. in Medicine, UNIFESP-EPM. Director of Service of Otorhinolaryngology, Hospital do Servidor Público do Estado de São Paulo; ^2^ Resident Physician, Service of Otorhinolaryngology, Hospital do Servidor Público do Estado de São Paulo; ^3^ Resident Physician, Service of Clinical Pathology, Hospital do Servidor Público do Estado de São Paulo; ^4^ Ph.D. in Medicine, University of Sao Paulo-FMUSP. Assistant Physician, Service of Otorhinolaryngology, Hospital do Servidor Público do Estado de São Paulo; Hospital do Servidor Público do Estado de São Paulo

**Keywords:** papilloma, squamous, middle ear

## Abstract

**S**quamous papillomas are benign neoplasms. The occurrence of middle ear squamous papilloma is rare. It is usually associated with nasosinusal pathology. The authors report a case of middle ear squamous papilloma and discuss its diagnostic aspects.

## INTRODUCTION

Squamous papilloma is a benign neoplasm derived from ectodermal tissue. These lesions occur normally on the skin of the face, trunk, arms and external auditory canal, which may affect the oral cavity, nose, pharynx and larynx mucosa, but rarely the middle ear^1^. According to Amado et al.^2^, the incidence of squamous papilloma within ENT organs is 2.47/100,000 inhabitants/year, affecting mainly the pharynx (9.51/100,000), oral cavity (6.87/100,000) and larynx (4.76/100,000), and are extremely rare in the middle ear (0.63/100,000). Middle ear papilloma is more frequent in female subjects as opposed to the male predominance of airway and external ear canal papillomas1., 2., 3., affecting in special subjects aged between 30 and 40 years.

Middle ear papilloma pathogenesis is uncertain, and it may be associated with human papilloma virus (HPV), but there is no correlation between it and genital disease1., 3., 4..

## CASE REPORT

**Figure 1:**
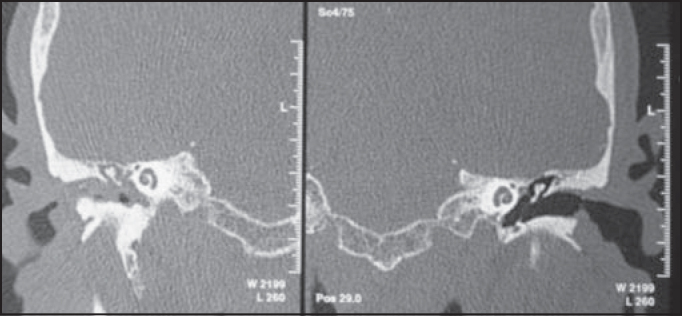
Temporal bone CT scan at coronal section evidencing complete obstruction of external auditory canal and right middle ear with soft part density and obliteration of oval and round window niches, preserved Chaussé spore. Left external and middle ears and both inner ears without any affections.

E.O.J, male, 72 years old, with history of right ear intermittent otorrhea, associated with ipsilateral hearing loss for 3 years. He did not report otalgia, otorrhagia, local trauma, vertigo or tinnitus. At otomycroscopy we identified lesion of polypoid multilobulated aspect with light pink transparent color comprising the whole right external auditory canal, coming from the middle ear. Left ear examination showed no affections. Temporal bone computer tomography scan (CT scan) evidenced complete obliteration of external and middle ears on the right with hyperattenuating material and evidence of ossicle chain erosion and obstruction of oval and round windows on the same side. The left ear did not present any affections (Figure 1).

After biopsy in the laboratory, under local anesthesia, it was possible to delimit the presence of wide central perforation of the tympanic membrane, and we could visualize residual lesions close to the malleus anterior process and posterior-superior region of tympanic membrane. The auditory tube orifice was free from the disease. There was resolution of otorrhea. Nasofibrolaryngoscopy did not show any abnormalities.

**Figure 2:**
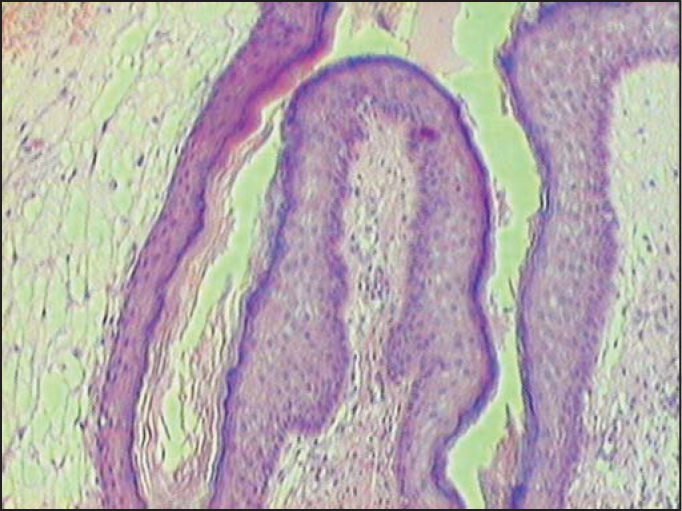
Histopathological examination of middle ear damage, HE stained cells, 400x. Dense connective axis with stratified paved keratinized epithelium without cell atypia.

Histopathology examination with sections stained with hematoxylin-eosin evidenced connective dense axis with pluri-stratified paved keratinized epithelium without cell atypia, with final diagnosis of squamous papilloma. HPV serology was negative (Figure 2).

Audiological assessment revealed presence of severe mixed hearing loss on the right with speech detection threshold at 90dB and moderate to severe sensorineural loss on the left and speech recognition index within the normal range.

**Figure 3:**
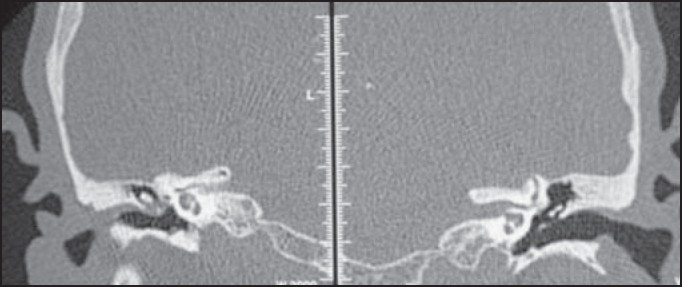
Temporal bone CT scan at coronal section evidencing partial obliteration of right middle ear and oval window niche with soft part density materials.

We performed CT scans that revealed the presence of material with attenuation of soft parts partially filling the right epitympanic cavity, as well as obliteration of the ipsilateral oval window niche with hypoattenuated and sclerotic mastoids (Figure 3).

The patient was submitted to radical mastoidectomy on the right and we observed that the mastoid was eburnean, with normal sized mastoid antrum, recovered with thick mucosa. Exposed to attic with removal of inflammatory tissue. The incus was normal and there was absence of the tip of malleus anterior process. We removed the border and tympanic annulus, as well as the whole thickened, edematous and friable mucosa of the promontorium and auditory tube. Postoperatively, there were no adverse events.

## DISCUSSION

Middle ear squamous papilloma is extremely rare1., 2.. The cause of the onset of this lesion is not well understood. Middle ear mucosa metaplasia by chronic inflammation may induce development of squamous papilloma. Ectopic migration of ectodermal tissue to the middle ear may also be implied in the genesis of this pathology4., 5.. Chao et al.^6^ showed association between HPV and cholesteatoma and middle ear papilloma without defining a causal correlation. However, Xia et al.^1^ demonstrated a dissemination of the squamous papilloma of nasopharynx to the tympanic membrane through the performance of myringotomy concomitantly with papilloma surgical treatment in the nasopharynx, indicating the likely infectious nature of the lesion. In the case reported, HPV test was negative.

Middle ear papilloma normally manifests with otorrhea and/or hearing loss, and there may be sensation of foreign body8., 9.. Clinical behavior of papilloma is variable, and it may be manifested with local aggressive growth, especially by inverted subtype, requiring mandatory clinical analysis of the lesion with biopsy for therapeutic planning. Temporal bone CT scan is essential to determine the extension of the lesion in the middle ear supporting the appropriate surgical planning.

An association that has been well documented is between middle ear papilloma and nasosinusal papilloma, especially in inverted subtype papilloma, making it necessary to investigate the nasal cavity, paranasal sinuses and nasopharynx7., 10., 11.. In the case reported here, nasofibrolaryngoscopy did not evidence any affection.

The treatment of middle ear papilloma is mainly surgical. It is necessary to perform complete resection of the lesion to prevent disease recurrence. Despite the appropriate treatment, in about 20% of the cases, there is lesion recurrence, and then it is necessary to perform new surgical interventions to be able to eradicate it^8^.

## CLOSING REMARKS

Middle ear squamous papilloma is a rare event. Despite the benign aspect of the lesion, the possibility of recurrence after surgical treatment makes it sometimes difficult to control this pathology. More studies are required to define its etiology.
